# Editorial: CRISPR-based genome editing for seed oil improvements in *Brassica napus* L.

**DOI:** 10.3389/fpls.2023.1273980

**Published:** 2023-08-25

**Authors:** Nazim Hussain, Rudolph Fredua-Agyeman

**Affiliations:** ^1^ Independent Researcher, Sharjah, United Arab Emirates; ^2^ Department of Agricultural, Food and Nutritional Science, University of Alberta, Edmonton, AB, Canada

**Keywords:** *Brassica napus* L., seed oil, fatty acids, mutation, breeding, CRISPR/Cas9, CRISPR-associated (Cas) proteins

Rapeseed (*Brassica napus* L., AACC, 2n = 38) is the world’s third-significant oilseed crop after soybean and oil palm, renowned for its high-quality edible oil and biofuel production (USDA ERS, 2021). The demand for rapeseed in various industries continues to surge, necessitating advancements in genetic traits to meet market requirements. Enhancing crop traits, both quantitatively and qualitatively, has always remained a focal point for agricultural researchers. Conventional and molecular approaches have been employed in the past; however, they are often time-consuming, lack precision and may result in genetic instability of desirable breeding traits. Recent advancements in genome editing technology, specifically Clustered Regularly Interspaced Short Palindromic Repeats (CRISPR) and CRISPR-associated (Cas) proteins, have revolutionized the field of plant breeding. This editorial delves into the potential of CRISPR technology in augmenting Brassica’s seed oil and fatty acid composition, as evidenced by publications featured in this Frontiers’ Research Topic titled “*CRISPR-Based Genome Editing for Seed Oil Improvements in Brassica napus L.*” By meticulously examining five publications, including one mini-review, and four research articles, this editorial aims to inspire researchers to embrace this revolutionary approach for rapeseed oil improvement and genetic enhancement. The insights presented here aim to emphasize CRISPR technology’s significance in empowering researchers towards achieving sustainable and enhanced agricultural practices. In this editorial, we summarize the key findings and perspectives outlined in each of the accepted articles.

## CRISPR/Cas9 technology for precision genome engineering

Ali and Zhang briefly reviewed CRISPR-mediated technology for seed oil improvement in rapeseed. Significant points are as follows: CRISPR/Cas9 technology has revolutionized genome editing by leveraging the Cas9 protein and single-guide RNAs (sgRNAs) to target specific regions of the genome (Cong et al., 2013; Lawrenson et al., 2015). In the case of rapeseed, an allotetraploid species with redundant genes (Chalhoub et al., 2014), CRISPR/Cas9 presents clear advantages over traditional breeding techniques. It enables simultaneous mutations in multiple copies of genes, making it a valuable tool for understanding polyploidy and achieving desired genetic improvements. Whether employing a single or multiplex genome editing strategy, the CRISPR/Cas9 system has proven effective for crop improvement (Lohani et al., 2020).

## Unraveling the role of STM in rapeseed development

The shoot apical meristem (SAM) is vital for plant growth, and studying its development can help create high-yield rapeseed breeds with desirable traits like multi-inflorescence structures and multilocular silique by editing the crucial genes in SAM (Yang et al., 2018; Xue et al., 2020; Lu et al., 2022). Thus, understanding SAM development is crucial for improving rapeseed varieties in agriculture. The SHOOT MERISTEMLESS (STM) gene, a transcription factor found in Arabidopsis, plays a vital role in SAM function and tissue boundary formation. However, the function of STM in rapeseed remains largely unexplored. In one of the articles published under the theme of our Research Topic, Yu et al. employed CRISPR/Cas9 technology to create single and double mutants of *BnaSTM* genes in rapeseed. The results unveiled the significance of *BnaA09.STM* and *BnaC09.STM* redundancy in regulating SAM development in rapeseed. Moreover, the findings shed light on the distinct role of *BnaSTM* in SAM maintenance compared to Arabidopsis. This breakthrough not only expands our understanding of rapeseed development but also highlights the potential of CRISPR technology in unraveling gene functions in polyploid crops.

## Enhancing seed oleic acid content through CRISPR/Cas9-mediated genome editing

Seed oleic acid content is a desirable trait in rapeseed breeding programs due to its health benefits (Terés et al., 2007; Terés et al., 2008) and impact on oil quality (Roszkowska et al., 2015; Cao et al., 2020; López et al., 2022). One of the studies under the current Research Topic by Liu et al. focused on the precise editing of the *BnFAD2* gene, responsible for seed oleic acid content, using CRISPR/Cas9 technology. By editing the double loci of *BnFAD2*, researchers were able to significantly increase the seed oleic acid content in rapeseed. The study meticulously evaluated editing efficiency, regeneration, and transformation rates, demonstrating the potential of CRISPR technology in enhancing seed oil traits. This breakthrough paves the way for the development of rapeseed varieties with improved oil quality through targeted genetic modifications.

## Portable diagnostic methods for identifying plant pathogens

Rapid and accurate detection of plant pathogens is crucial for preventing the spread of diseases and ensuring crop health. *Leptosphaeria maculans* (*L. maculans*), a fungus responsible for phoma stem canker disease in rapeseed, causes substantial yield losses (Rouxel and Balesdent, 2005; Fitt et al., 2006). Lei et al. developed a portable detection method that combines CRISPR/Cas12a-based detection with recombinase polymerase amplification (RPA). This innovative approach allows for on-site detection of *L. maculans*, enabling timely disease management decisions. The integration of CRISPR technology in plant pathology diagnostics represents a significant advancement in disease surveillance and control strategies.

## Enhancing MUFA content: a challenge and opportunity

Our Research Topic also explores recent advancements in genetic engineering to address the challenge of low Monounsaturated fatty acids (MUFAs) levels in vegetable oils. MUFAs, like oleic acid, are favored in both industrial (biodiesel fuels and biolubircants), and food applications due to their higher thermal-oxidative stability and viscosity compared to other fatty acids (Smith et al., 2007; Davis et al., 2008). However, most vegetable oils have high Polyunsaturated fatty acids (PUFA) and low MUFA levels. To tackle this, Lee et al. used CRISPR-Cas9-mediated gene editing with one single guide RNA (sgRNA) to create a triple *CsFAD2 KO* Camelina (a genus in the Brassicaceae family) mutant plants. The mutant successfully increased MUFA content in Camelina seeds but at the cost of inhibited growth and compromised agronomic traits. So, achieving MUFA levels above 80% while maintaining a normal phenotype has proven to be a challenging endeavor.

The researchers, Lee et al., propose a multi-pronged approach to circumvent the growth limitations associated with increased monounsaturated fatty acid (MUFA) content in Camelina seeds. Firstly, they suggest targeting the gene(s) responsible for polyunsaturated fatty acid (PUFA) accumulation in addition to the *CsFAD2* knockout mutation to further enhance MUFA levels. This would create a *CsFAD2* double mutant form (aa/bb/CC) where PUFA-related genes are mutated alongside *CsFAD2*, achieving a balance between increased MUFA content and normal growth. Another approach they suggest is overexpressing the gene(s) involved in PUFA biosynthesis within the chloroplasts of the *CsFAD2* triple mutant form (aa/bb/cc). This strategy could lead to the development of Camelina varieties with enhanced MUFA content while maintaining the desired agronomic traits necessary for robust growth and development. Successful implementation of these strategies not only expands the applications of Camelina seed oil but also enables the production of unconventional fatty acids derived from MUFAs, such as hydroxy fatty acids, which could be valuable precursors for various industrial purposes.

## Challenges and future prospects

The studies discussed in this editorial exemplify the power of CRISPR technology in enhancing rapeseed’s oil quality, unraveling gene functions, and developing portable diagnostic methods for disease management. While the potential of CRISPR technology for rapeseed improvement is promising, challenges and ethical considerations remain. Off-target effects, regulatory hurdles, and public acceptance are among the key challenges faced by researchers. Addressing these concerns requires stringent guidelines, thorough risk assessment, and effective communication between scientists, policymakers, and the public. Furthermore, optimizing CRISPR delivery systems, improving editing efficiency, and expanding targetable genetic elements are crucial for the future success of CRISPR-mediated rapeseed improvement.

## Author contributions

NH: Conceptualization, Supervision, Validation, Writing – original draft, Writing – review & editing. RF: Validation, Writing – review & editing.
